# Stimulator of interferon response cGAMP interactor overcomes ERBB2-mediated apatinib resistance in head and neck squamous cell carcinoma

**DOI:** 10.18632/aging.203475

**Published:** 2021-08-30

**Authors:** Guo Ye, Junbin Zhang, Chengyao Zhang

**Affiliations:** 1 Department of Head and Neck Cancer Center, Chongqing University Cancer Hospital, Chongqing 400030, China; 2 Chongqing Key Laboratory of Translational Research for Cancer Metastasis and Individualized Treatment, Chongqing University Cancer Hospital, Chongqing 400030, China

**Keywords:** head and neck squamous cell carcinoma, apatinib resistance, STING, ERBB2, proliferation

## Abstract

Purpose: Apatinib resistance is the main obstacle to the effective treatment of advanced head and neck squamous cell carcinoma (HNSCC). This study aimed to evaluate the function of Erb-B2 receptor tyrosine kinase 2 (ERBB2) and stimulator of interferon response cGAMP interactor (STING) in apatinib resistance in HNSCC.

Method: The Cancer Genome Atlas database of HNSCC was used to analyze the relationship between vascular endothelial growth factor receptor 2 (VEGFR2) expression and prognosis. An apatinib resistant (AR) HNSCC cell line was constructed based on the CAL27 cell line. RNA sequencing was performed to explore the differentially expressed mRNAs. Quantitative real-time reverse transcription PCR (qRT-PCR) and western blotting were used to evaluate the expression and phosphorylation level VEGFR2, ERBB2, STING, and related proteins. Apatinib resistance was evaluated by colony formation and cell viability assays. A mouse subcutaneous tumor formation model was established to evaluate the efficiency of combination treatment and vascularization was evaluated by assessing CD31 immunofluorescence.

Result: The expression of VEGFR2 was high in tumor of patients with HNSCC. Western blotting and qRT-PCR revealed that in AR cells, ERBB2 expression was high, whereas the expression of STING was low. Targeted treatment of ERBB2 using lapatinib could attenuate apatinib resistance. Further research confirmed that overexpressing *STING* could decrease ERBB2 expression.

Conclusion: STING could sensitize AR cells to apatinib by decreasing ERBB2 expression. The combination of lapatinib or a STING agonist with apatinib ameliorated acquired apatinib resistance in a synergistic manner.

## INTRODUCTION

Head and neck squamous cell carcinoma (HNSCC), as one of the most common malignant carcinomas, has a poor prognosis, as indicated by its high recurrence rate and metastasis risk. The 5-year overall survival (OS) of patients with HNSCC is 40–50%, while that of patients with advanced stage disease is 30–40% [1, 2]. Local and distant tumor recurrence are the main causes of death in patients with locally advanced HNSCC.

Angiogenesis, the process by which pre-existing blood vessels form new capillaries, is a crucial biological process in normal physiology, for example in healing wounds. In addition, angiogenesis is important in pathological conditions, for example in accelerating the growth, progression, and metastasis of tumors [3, 4]. Various pro-angiogenic signaling pathways drive angiogenesis, among which the vascular endothelial growth factor (VEGF)/VEGF receptor (VEGFR) pathway is the most important [5, 6].

Apatinib is an oral tyrosine kinase inhibitor of VEGFR2, which exhibited promising anti-neoplastic and antiangiogenic and activities in certain tumors, such as breast carcinoma [7], sarcoma [8], hepatocellular carcinoma [9], non-small-cell lung cancer [10], and recurrent epithelial ovarian cancer [11]. Although apatinib reverses multidrug resistance of chemotherapeutic agents, apatinib resistance can occur, making its full use challenging [8]. For some VEGFR inhibitors, the duration of response is short before the drug resistance occurs, resulting in only limited improvements in progression-free survival (PFS) and OS. Given apatinib’s central role in targeted therapy and the unsatisfactory clinical outcome resulting from apatinib resistance, new methods to ameliorate apatinib resistance are required [12].

In the present study, overexpression of the oncogene *ERBB2* (encoding Erb-B2 receptor tyrosine kinase 2), and low expression of the antioncogene *STING* (encoding stimulator of interferon response cGAMP interactor), were observed in apatinib resistant (AR) cells. Accordingly, we hypothesized that STING and ERBB2 might regulate apatinib resistance in HNSCC via unknown pathway. The relationship between prognosis and VEGFR2 expression was first evaluated. The expression and function of STING and ERBB2 in apatinib resistance of HNSCC were further evaluated. The STING/ERBB2 pathway provides a potential target to overcome apatinib resistance during VEGFR2 inhibition therapy.

## RESULTS

### Highly expressed VEGFR2 induces angiogenesis in HNSCC

**Figure 1:**
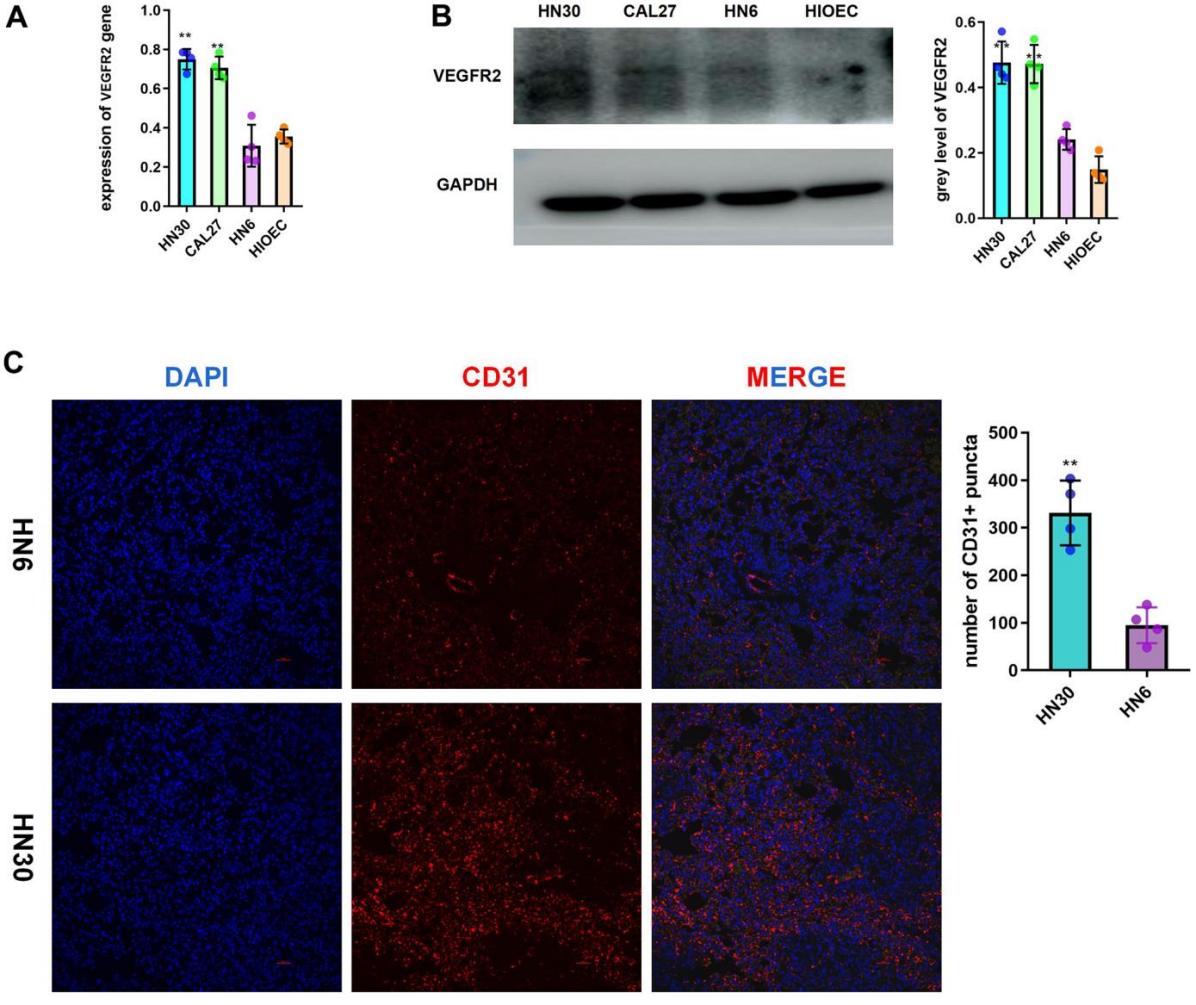
**Highly expressed VEGFR2(KDR) induces angiogenesis in HNSCC.** (**A**, **B**) qRT-PCR and western blotting results for VEGFR2 in HIOEC and HNSCC cell lines (HN30, CAL27, and HN6) (**C**) Representative images of HN6 and HN30 subcutaneous tumors using immunofluorescence staining against CD31 and DAPI staining of nuclei. Higher angiogenesis was observed in the HN30 group, which has a higher VEGFR2 expression level. *P < 0.05, **P < 0.01, ***P < 0.001 versus the control.

According to the analysis of the TGCA-HNSC dataset, *VEGFR2* (also known as *KDR*) expression was increased in cancer tissue compared with that in normal tissue. (Supplementary Figure 1A). Compared with that of the patients with low *VEGFR2* expression, the survival time of patients with high *VEGFR2* expression was shorter (Supplementary Figure 1B). According to the qRT-PCR and western blotting results, HNSCC cells had higher VEGFR2 levels than HIOECs. VEGFR2 expression was lower in HN6 cells than in HN30 and CAL27 cells (Figure 1A, 1B). In accordance with the *in vitro* results, subcutaneous tumors formed by HN30 showed a greater degree of angiogenesis than of those formed by HN6 cells (Figure 1C).

### High ERBB2 expression and low STING expression were observed in AR cells

**Figure 2:**
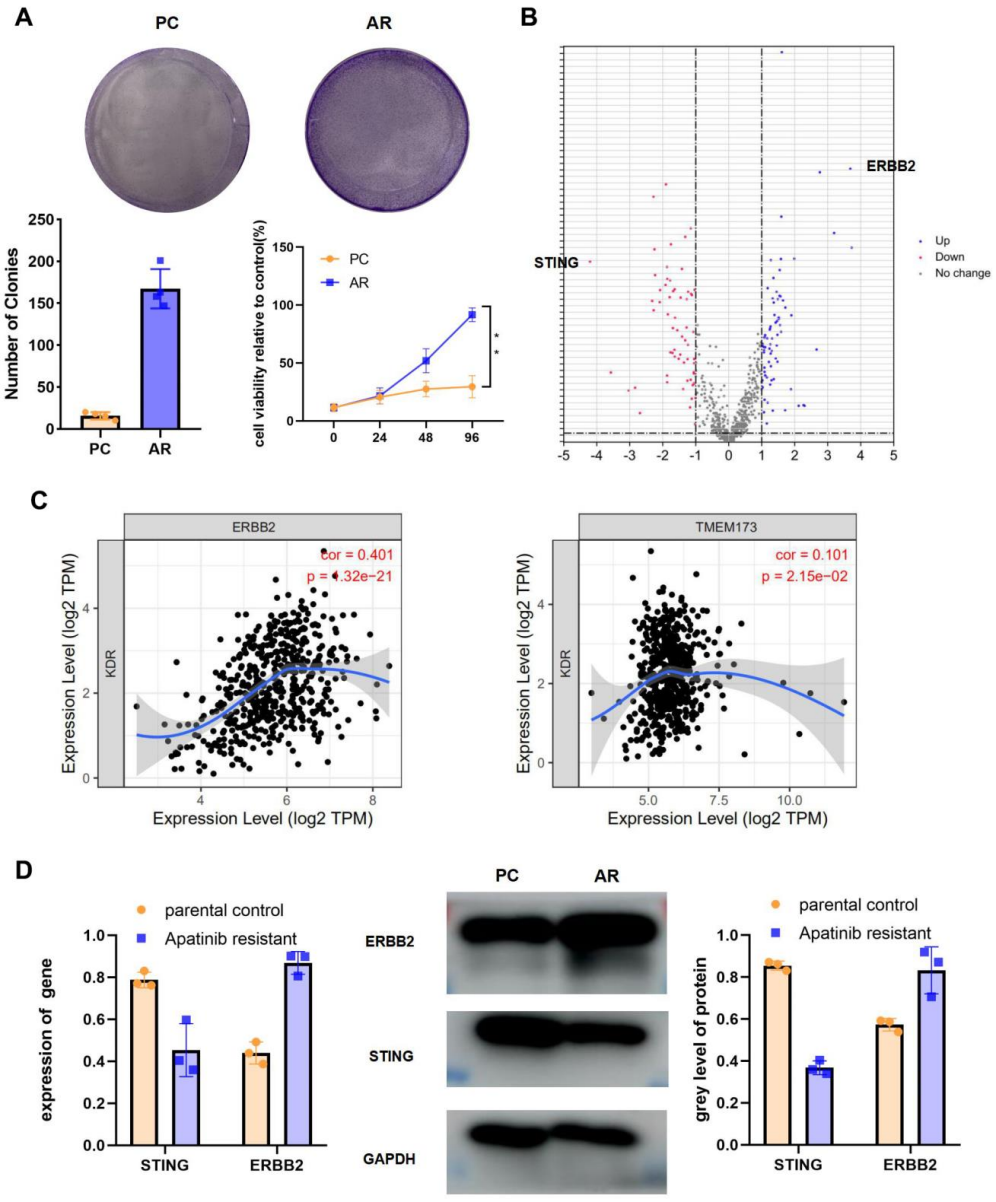
**High ERBB2 expression and low STING expression were observed in AR cells.** (**A**) Cell proliferation after treatment with apatinib for different times, as assessed using an MTT assay (*P < 0.05, **P < 0.01). The successful establishment of AR HN30 cells was demonstrated by their insensitivity to apatinib. Colony formation of AR cells was enhanced compared with that of the control. (**B**) Venn diagram of predicted up or downregulated mRNAs for AR cells compared with PC controls. A total of 198 downregulated and 277 upregulated mRNAs were obtained for differential analysis. *STING* and *ERBB2* were identified as having the highest fold change. (**C**) A correlation was determined among *VEGFR2*, *ERBB2*, and *STING* using TIMER correlation analysis (http://timer.cistrome.org/). (**D**) AR and PC cells were treated with apatinib (20 μM) for 24 hours. Then, qRT-PCR and western blotting were performed to assess expression of ERBB2 and STING in AR and PC cells. *P < 0.05, **P < 0.01, ***P < 0.001 versus the control.

The colony formation assay and CCK-8 results for the AR cells compared with the PC cells showed that proliferation occurred in a fold-change manner (Figure 2A). (**P < 0.01). The viability of the AR cells higher than that of the PC controls. To analyze the signaling pathways related to apatinib resistance, RNA seq of AR and PC cells was performed. A total of 198 downregulated and 277 upregulation mRNAs were obtained for differential analysis (Figure 2B). *STING* and *ERBB2* showed the highest fold change. To evaluate the relationship between VEGFR2, STING, and ERBB2, the TIMER database was used (http://timer.cistrome.org/), which identified a negative correlation between *VEGFR2* and *STING* (also known as *TMEM173*) (Figure 2C). Meanwhile, a positive correlation was confirmed between VEGFR2 and ERBB2 (Figure 2C). In accordance with bioinformatic results, qRT-PCR and western blotting results showed upregulation of ERBB2 levels and downregulation of STING levels in AR cells compared with those in PC cells (Figure 2D).

### An ERBB2 inhibitor, lapatinib, and apatinib in combination re-sensitized AR cells to apatinib

**Figure 3:**
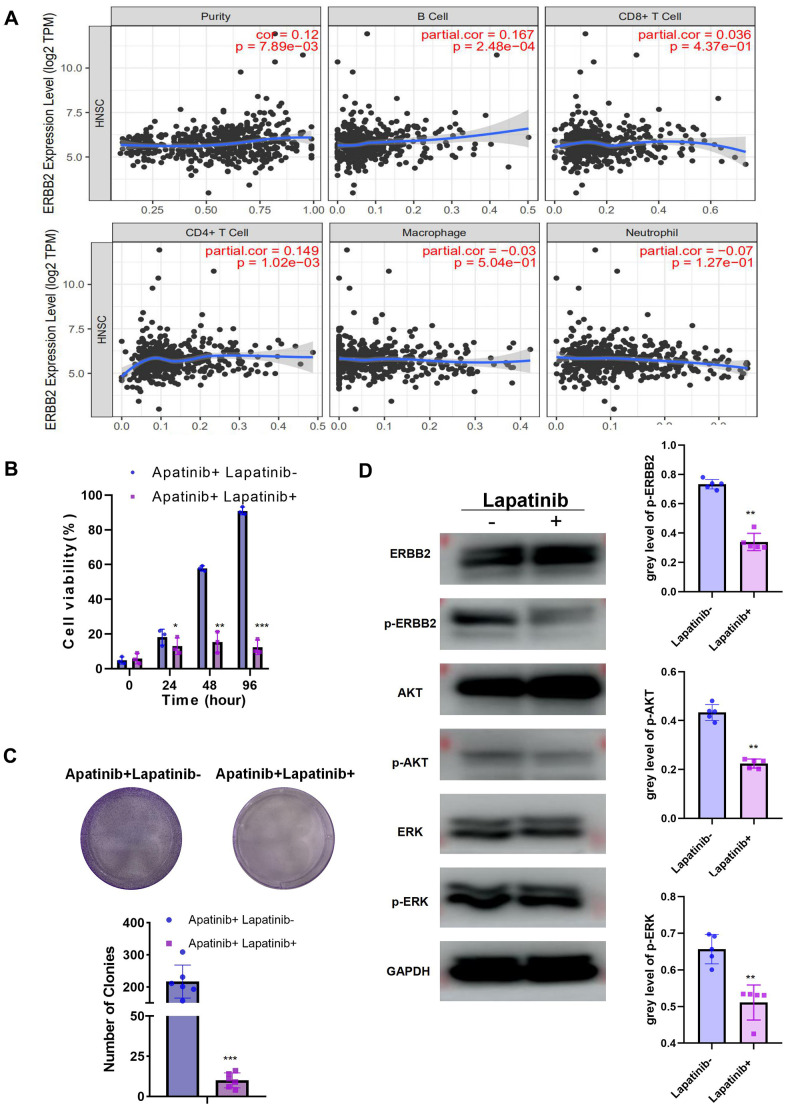
The combination of lapatinib and apatinib re-sensitizes AR cells to apatinib (**A**) The negative correlation between ERBB2 and TILs was confirmed using TIMER 2.0. (**B**) Cell viability after apatinib (20 μM) treatment, with or without lapatinib (20 μM), for different times, as assessed using an MTT assay. The combination of lapatinib and apatinib re-sensitized AR cells to apatinib. (**C**) Colony formation was inhibited in the combination group compared with that in the groups treated with each drug alone. (**D**) Western blotting illustrating the abundance of related signaling pathway proteins after apatinib (20 μM) treatment, with or without lapatinib(20 μM). Lapatinib treatment suppressed the levels of ERRB2, AKT and ERK phosphorylation. *P < 0.05, **P < 0.01, ***P < 0.001 versus the control.

To further explore the function of ERBB2 in apatinib resistance, TIMER analysis was used, which revealed the negative correlation between ERBB2 and immune cell infiltration (Figure 3A). This led us to speculate that apatinib combined with an ERBB2 inhibitor might increase apatinib sensitivity effectively. Lapatinib is an FDA approved ERBB2 inhibitor. According to a previous report [13], phosphorylation of ERBB2 is greatly downregulated by lapatinib. To address the efficacy of ERBB2 inhibition in apatinib resistance, AR cells were treated with lapatinib combined with apatinib. The results showed that the combined treatment suppressed AR cell proliferation in a synergistic manner (Figure 3B, 3C). Lapatinib has been reported to inhibit the growth of cancer via the ERBB2/AKT/mTOR [14] and RAF/MEK/ERK [15, 16] signaling pathways. The western blotting results showed that levels of p-ERBB, p-ERK, and p-AKT in AR cells decreased after 24 h of treatment with apatinib and lapatinib (Figure 3D). The amount of total AKT or ERBB2 protein in AR cells did not change after treatment.

### Phosphorylation of ERBB2 could be inhibited by a STING agonist, vadimenzan

**Figure 4:**
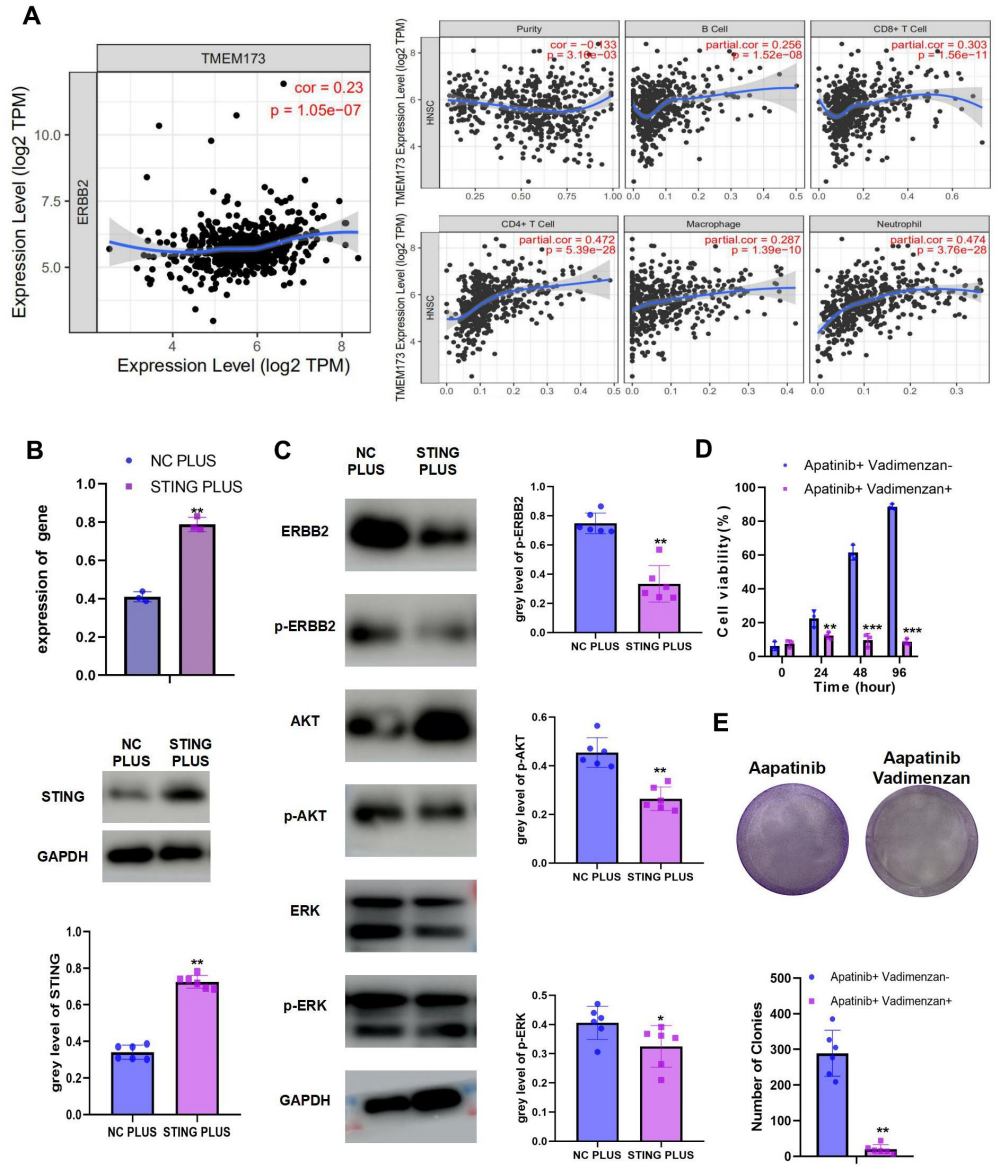
The combination of STING and apatinib re-sensitizes AR cells to apatinib (**A**) The positive correlation between STING and TILs was confirmed using TIMER 2.0. (**B**) AR cells were transfected with a STING overexpression plasmid, qRT-PCR and western blot result confirmed the successful establishment of STING PLUS AR cells. (**C**) Levels of ERBB2, AKT, ERK, p-ERBB2, p-AKT, and p-ERK were detected using western blotting. Compared with NC PLUS cells, the levels of ERBB2, p-ERBB2, p-AKT, and p-ERK were downregulated in STING PLUS cells. (**D**) MTT assay illustrating the cell viability of NC PLUS and STING PLUS AR cells after apatinib (20 μM) treatment for different times. The combination of STING and apatinib re-sensitized AR cells to apatinib. (**E**) Colony formation was inhibited in the combination group compared with that in the groups treated with each drug alone. *P < 0.05, **P < 0.01, ***P < 0.001 versus the control.

We observed a negative correlation between ERBB2 and STING (Figure 4A). Moreover, STING could stimulate immune cell infiltration. To explore the mechanism of STING in ERBB2-mediated apatinib resistance, a plasmid overexpressing *STING* was constructed and transfected into AR cells (Figure 4B). STING overexpression was confirmed using western blotting and qRT-PCR. Overexpression of *STING* resulted in inhibition of the phosphorylation of ERBB2, AKT, and ERK (Figure 4C). Considering the significance of STING in apatinib resistance, we hypothesized that the combination of apatinib with a STING agonist, Vadimenzan, would effectively enhance apatinib sensitivity. Combined treatment with vadimenzan and apatinib displayed a synergistic effect by markedly inhibiting AR cell proliferation (Figure 4D, 4E).

### The combination of lapatinib and vadimenzan re-sensitizes AR cells to apatinib *in vivo*

**Figure 5:**
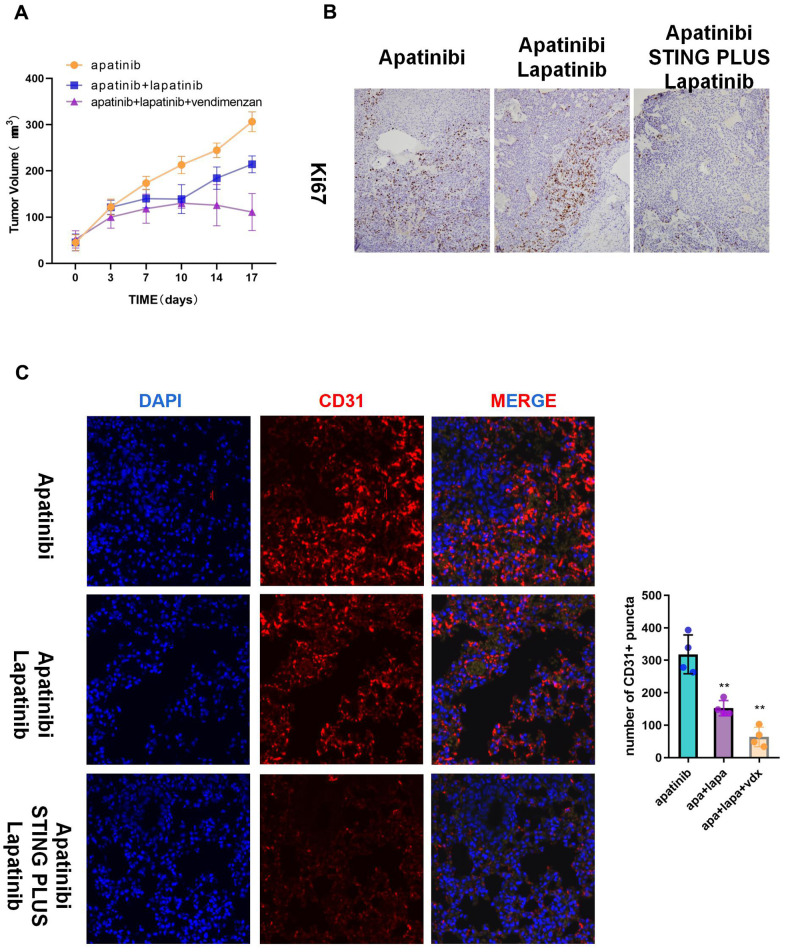
**The combination of vadimenzan and lapatinib re-sensitizes AR cells to apatinib *in vivo*.** (**A**) After washing with PBS three times, AR cells (total of 1 × 10^6^) suspended in 50 μL of DMEM were injected in each injection site. The tumor volumes of the mice were evaluated among the three groups. (**B**) Ki67 staining images of cancer samples in the different groups. (**C**) Representative images of subcutaneous tumors using immunofluorescence staining against CD31 and DAPI nuclear staining. *P < 0.05, **P < 0.01, versus the control.

We used HNSCC xenografts in nude mice to verify the *in vitro* results. The tumor volume in the combined treatment group was significantly smaller than that in the groups treated with each single agent (Figure 5A). Moreover, immunohistochemistry indicated decreased Ki67 levels, which suggested inhibition of proliferation in the combined treatment group (Figure 5B). Correspondingly, the subcutaneous tumors of the combination group showed less angiogenesis than those from the apatinib only group (Figure 5C).

## DISCUSSION

Therapeutic strategies targeting vascular endothelial growth factor receptors (VEGFRs) have been studied extensively because of the important roles of VEGFRs in carcinogenesis [17]. Apatinib is an oral tyrosine kinase inhibitor of VEGFR-2 that can induce autophagy [18] and apoptosis, and suppresses tumor proliferation in anaplastic thyroid cancer [8], hepatocellular carcinoma [19], and osteosarcoma [20]. However, effective treatment is challenged by apatinib resistance [21]. The acquired resistance involves the activation of pathways such as JAK/STAT, PI3K/AKT, and MAK/ERK signaling [22, 23]. Thus, new approaches are required to enhance apatinib’s efficacy [7, 24]. However, most related research has focused on microRNAs and circular RNAs, which are unlikely to be transformed into clinical application in coming years. ERBB2 is a well-known oncogene, and in preclinical studies of HER2-positive advanced solid tumors, ERBB2 inhibitors have displayed very good antitumor activity, both *in vivo* and *in vitro* [25, 26]. A previous study showed that an ERBB2 inhibitor combined with apatinib was effective against HER2-positive gastric cancer and acquired resistance against the ERBB2 inhibitor [27]. In HNSCC, ERBB2 is upregulated in primary and metastatic tumors, which is related to poor prognosis [28].

In this study, we confirmed that upregulation of ERBB2 enhances apatinib resistance through PI3K/AKT and MAK/ERK signaling, which is consistent with the results of previous studies [22, 23]. We speculated that phosphorylation of ERBB2 could increase apatinib resistance by activating AKT and ERK. Inhibition of ERBB2 phosphorylation by the TKI inhibitor, lapatinib, effectively re-sensitized AR cells to apatinib. Furthermore, cell viability was significantly decreased under the combined treatment of apatinib and lapatinib.

STING expression correlates negatively with that of many oncogenes and is thus believed to be a tumor suppressor [29, 30]. Within the tumor microenvironment (TME), STING pathway activation in antigen-presenting cells leads to increased production of type I interferons and promotes tumor-mediated cross priming of CD8+ T cells, finally resulting in adaptive anticancer immune responses [31, 32]. STING shows strong expression in HPV+ HNSCC cancer cells, but not in HPV^−^ HNSCC cancer cells [33]. STING ligands administered locally to the tumor led to non-autonomous activation of STING in non-cancer cells in the TME, suggesting that such therapy might be effective to treat STING^−^ and STING^+^ tumors [33, 34]. According to these activities, STING agonists were demonstrated to synergize cancer treatment by promoting CAR T cells or overcoming tumor resistance to PD-1 blockade [35–37]. Our research has shown that the antitumor effort of STING acts via proliferation and this proliferation sensitizes AR cells to apatinib. To the best of our knowledge, this is the first report of the effect of STING on apatinib resistance.

In HPV^-^ HNSCC, STING expression is suppressed, and is further downregulated in AR cells; therefore, we investigated whether treatment promoting STING combined with apatinib has therapeutic potential in HNSCC. Our results demonstrated that ERBB2, AKT, and ERK confer apatinib desensitization. In addition, vadimenzan, a STING agonist, attenuated apatinib desensitization and decreased the proliferation on AR cells *in vitro*. *In vivo*, vadimenzan treatment resulted in xenografts becoming sensitive to apatinib therapy. Thus, targeting the ERBB2/AKT/ERK axis by stimulating STING represents a feasible method to restore the apatinib sensitivity of HNSCC cells.

Overall, the results of the present study indicated that the ERBB2/AKT/ERK axis regulates apatinib desensitization. Importantly, upregulation of STING expression overcame apatinib resistance effectively by inhibiting ERBB2 phosphorylation. Apatinib combined with a STING agonist, e.g., vadimenzan, could be used to ameliorate apatinib resistance in HNSCC.

## MATERIALS AND METHODS

### Cells and chemicals

The American Type Culture Collection (ATCC) (Manassas, VA, USA) provided the CAL27, HN6, and HN30 cells. Human immortalized oral epithelial cells (HIOECs) were grown in defined keratinocyte serum-free medium (Invitrogen, Waltham, MA, USA). CAL27, HN6, and HN30 cells were grown in Dulbecco’s modified Eagle’s medium (DMEM) (Gibco, Grand Island, NY, USA) containing 1% penicillin-streptomycin, 1% glutamine, and 10% fetal bovine serum. Apatinib was obtained from Hengrui Medicine Co., Ltd. (Jiangsu, China). The STING agonist, vadimenzan, was purchased from Selleck CO., Ltd. (Shanghai, China). *In vitro*, AR CAL27 cells were established by treating the cells with 5 μM apatinib initially and the increasing the concentration incrementally to 20 μM once a week for 3 months. To verify the successful establishment of AR cells, 3-(4,5-dimethylthiazol-2-yl)-2,5-diphenyltetrazolium bromide (MTT) assays were performed.

### Bioinformatics

The mRNA profiles (Normal: 44, Tumor: 520) were obtained from The Cancer Genome Atlas (TCGA) database as the TCGA-HNSC dataset (https://portal.gdc.cancer.gov/). Kaplan–Meier analysis of OS and PFS was performed based on the TCGA-HNSC dataset.

### Quantitative real-time reverse transcription PCR (qRT-PCR)

The total mRNAs were extracted from HIOEC and HNSCC cells lines. A NanoDrop 2000/2000C spectrophotometer (Nanodrop Technologies, Wilmington, DE, USA) was used to assess the RNA purity and concentration at wavelengths of 260/280 nm. The RNA was transcribed into cDNA using a PrimeScript™ RT Reagent Kit (Takara Biotechnology, Dalian, China). A TB Green® Premix Ex TaqTM Kit (Takara Biotechnology) master mix was used to perform the qPCR reactions using the cDNAs as templates on a StepOnePlus™ Real-Time PCR System (Applied Biosystems, Foster City, CA, USA). *GAPDH* (encoding glyceraldehyde-3-phosphate dehydrogenase) expression was detected as an internal control. The primers used for qPCR of human genes were:

**Table:** 

**Gene name**	**Type**	**Sequence 5'-3'**
*ERBB2*	Forward	TGCAGGGAAACCTGGAACTC
	Reverse	ACAGGGGTGGTATTGTTCAGC
*STING*	Forward	CGCTTCCTGGATAAACTGCC
	Reverse	GCCCACAGTAACCTCTTCCT
*VEGFR2*	Forward	GGCCCAATAATCAGAGTGGCA
	Reverse	CCAGTGTCATTTCCGATCACTTT
*GAPDH*	Forward	AATCCCATCACCATCTTCCAG
	Reverse	GAGCCCCAGCCTTCTCCAT

### Western blotting

Cells were treated with 20 μM apatinib, with or without lapatinib (20 μM) and vadimenzan (20 μM), for 24 h. Then, at various time points, we extracted total cellular proteins. The proteins were separated electrophoretically and then electrotransferred onto a membrane. Next, 5% skim milk in 1% TBST was used to block the membrane for 1 h at 4° C. The proteins on the membrane were then reacted with primary antibodies that recognized STING (catalog number 13647S; 1:1,000); ERBB2 (catalog number 2165S; 1:1,000); phosphorylated (p)-ERBB2 (catalog number 6942; 1:1,000); VEGFR2 (catalog number 9698; 1:1,000); protein kinase B (AKT) (catalog number 4685S; 1:1,000); p-AKT (catalog number 4060S; 1:1,000); extracellular regulated kinase (ERK) (catalog number 9194S; 1:1,000); p-ERK (catalog number 4370S; 1:1,000); and GAPDH (catalog number 174S; 1:1,000). All primary antibodies were purchased from Cell Signaling Technology (Danvers, MA, USA).

### Immunohistochemistry and immunofluorescence

To evaluate angiogenesis, an anti-CD31 primary antibody was incubated with tumor sections, followed by reaction with an Alexa 488-conjugated goat anti-mouse secondary antibody according to the manufacturer’s protocol. 4′,6-diamidino-2-phenylindole (DAPI) was used to stain the cell nuclei. The stained sections were observed under a confocal microscope (SP5, Leica, Wetzlar, Germany). To evaluate cell apoptosis, marker of proliferation Ki-67 (Ki67) staining was performed using anti-Ki67 primary antibodies.

### Colony formation and cell viability assays

To assess cytotoxicity, parental control (PC), AR, and CAL27 cells were seeded at a density of 1 × 10^4^ cells/ml in 96-well flat-bottom plates in triplicate and cultured in 100 μL medium for 12 h before being exposed to apatinib, with or without lapatinib and vadimenzan. Then, Cell Counting Kit 8 (CCK-8) solution (10% in 100 μL of medium; Dojindo, Japan) was added to each well at different timepoints. The absorbance at 450 nm was measured after 2 h of incubation. For the colony formation assay, PC, AR, and CAL27 cells were seeded in six-well plates at 1 × 10^4^ cells per well. Ten days later, neutral paraformaldehyde was used to fix the cells, followed by staining with a crystal violet solution. Formed colonies comprising 50 to 100 cells were counted.

### RNA sequencing (RNA-seq)

For apatinib resistance studies, the HiseqXTen system (Genomeditech Co. Ltd., Shanghai, China) was used to perform RNA-seq. Differentially expressed mRNAs were identified based on their fold-change in expression and their P-values, which were determined using one-way analysis of variance. Differentially expressed genes (DEGs) were displayed on a volcano plot according the set as a fold-change of X and a P-value of Y, and the DEGs were displayed used a volcano plot.

### Tumor xenografts

The Chongqing experimental animal center supplied 4-week-old specific pathogen free male BALB/c nude mice (nu/nu), weighing 16.31 ± 0.9 g. All laboratory procedures were approved by the laboratory animal care and use committee of the hospital. HN6/HN30 cells (1 × 10^6^ cells) were washed in PBS three times before being suspended in 50 μL of DMEM per injection site. The cells were injected subcutaneously into the back of the right rear leg in each group of mice (n = 5). The sample size was calculated according to previous research [38, 39]. At 10 days after injection, the average tumor volume was nearly 200 mm^3^. At this point, the mice were euthanized humanely. In another experiment, 1 × 10^6^ AR cells (3rd passage) were washed in PBS three times before being suspended in 50 μL of DMEM per injection site. The cells were injected subcutaneously into the back of the right rear leg of the mice. Seven days later, the average tumor volume was nearly 125 mm^3.^ The exact sizes of the tumors on each animal before treatment are shown in Supplementary Table 1. Then, the mice were randomly separated into three groups (n = 5 per group): the apatinib group (orally-delivered apatinib at 150 mg/kg per day), the apatinib+lapatinib group (the same dose and schedule of apatinib plus lapatinib solution (100 mg/kg)), and the apatinib+lapatinib+vendimenzan group (the same oral doses and schedule of apatinib and lapatinib plus i.p. administered vadimenzan (50 mg/kg) twice a week). The development and progression of solid tumors were monitored every two days until the tumor reached greater than 1.5 cm in length. At this point, the mice were euthanized humanely. The tumor volume (V) was calculated as: V = L x W^2^/2, where L is the tumor length and W is the tumor width. Immunohistochemistry and immunofluorescence analyses were performed on xenograft samples.

### Statistical analysis

The mean ± SD was used to represent continuous variables. The clinical and histological data were analyzed using the chi-squared test or Pearson’s chi-squared test. All statistical data analysis was carried out using GraphPad Prism version 7 (GraphPad Software Inc., San Diego, CA, USA). The statistical significance of differences was assessed using Student's t-test and one-way analysis of variance. In the figures, statistical significance is indicated using: *p < 0.05, **p < 0.01 and ***p < 0.001.

### Ethics approval and consent to participate

The Ethical Committee of Chongqing University Cancer Hospital approved the study design.

## Supplementary Material

Supplementary Figure 1

Supplementary Table 1
