# Supplementary material for: Stimulator of interferon response cGAMP interactor overcomes ERBB2-mediated apatinib resistance in head and neck squamous cell carcinoma
Source: Aging (Albany NY). 2021 Aug 30;13(16):20793–807. doi: 10.18632/aging.203475 (PMC8436913; doi:10.18632/aging.203475)
Supplement: Supplementary Figure 1 [file aging-13-203475-s001.pdf]

# SUPPLEMENTARY FIGURE

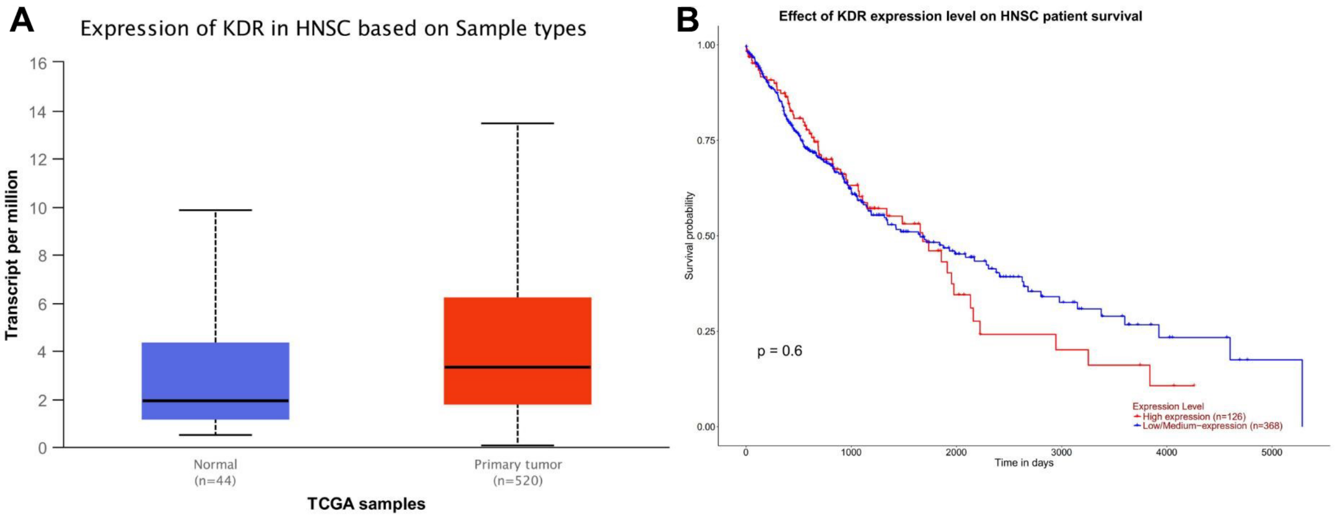


**Supplementary Figure 1.** (**A**) Compared with the normal group (blue), the expression of VEGFR2 was high in tumor samples (red); (**B**) Survival curves of VEGFR2 expression for prognosis in the TCGA-HNSC dataset. Red represents the high expression group and blue represents the low expression group.
