# Supplementary material for: Stimulator of interferon response cGAMP interactor overcomes ERBB2-mediated apatinib resistance in head and neck squamous cell carcinoma
Source: Aging (Albany NY). 2021 Aug 30;13(16):20793–807. doi: 10.18632/aging.203475 (PMC8436913; doi:10.18632/aging.203475)
Supplement: Supplementary Table 1 [file aging-13-203475-s002.pdf]

SUPPLEMENTARY TABLE

Supplementary Table 1. The exact sizes of the tumors on each animal before treatment.

Group	Tumor volume (mm^3)				
Apatinib	130	123	171	148	145
apatinib+lapatinib	145	128	123	150	174
apatinib+lapatinib+vendimenzan	171	148	174	150	123
